# A microencapsulation approach to design microbial seed coatings to boost wheat seed germination and seedling growth under salt stress

**DOI:** 10.3389/fpls.2023.1283590

**Published:** 2023-11-21

**Authors:** Min Gong, Jiuxing He, Meng Kong, Qiuyan Huo, Yawen Jiang, Jiqing Song, Wei Han, Guohua Lv

**Affiliations:** ^1^ Institute of Environment and Sustainable Development in Agriculture, Chinese Academy of Agricultural Sciences, Beijing, China; ^2^ Shandong Agro-tech Extension Center, Jinan, China; ^3^ Institute of Dongying Shengli Salt Alkali Agriculture Industrialization and Technology Research, Dongying, China

**Keywords:** bacteria, microcapsules, salt stress, seed coating, wheat

## Abstract

**Introduction:**

Salt stress in seed germination and early seedling growth is the greatest cause of crop loss in saline-alkali soils. Microbial seed coating is an effective way to promote plant growth and salt resistance, but these coatings suffer from poor seed adhesion and low survival rates under typical storage conditions.

**Methods:**

In this study, the marine bacterium *Pontibacter actiniarum DSM 19842* from kelp was isolated and microencapsulated with calcium alginate using the emulsion and internal gelation method.

**Results:**

Compared to unencapsulated seeds, the spherical microcapsules demonstrated a bacterial encapsulation rate of 65.4% and survival rate increased by 22.4% at 25°C for 60 days. Under salt stress conditions, the seed germination percentage of microcapsule-embedded bacteria (M-Embed) was 90%, which was significantly increased by 17% compared to the germination percentage (73%) of no coating treatment (CK). Root growth was also significantly increased by coating with M-Embed. Chlorophyll, peroxidase, superoxide dismutase, catalase, proline, hydrogen peroxide and malondialdehyde levels indicated that the M-Embed had the best positive effects under salt stress conditions.

**Discussion:**

Therefore, embedding microorganisms in suitable capsule materials provides effective protection for the survival of the microorganism and this seed coating can alleviate salt stress in wheat. This process will benefit the development of sustainable agriculture in coastal regions with saline soils.

## Introduction

Biotic and abiotic stressors in the environment have become major factors limiting global crop growth and production and increased soil salinization has become a primary factor that decreases growth and production (Tavakkoli et al., 2011; Abideen et al., 2020). Saline environments harm plants through physiological drought (Dantas et al., 2019), ion dysregulation (Habib et al., 2016) and, oxidative stress (Yang and Guo, 2018) and leads to a generated metabolic disorder (Zhang et al., 2021). Seed germination and seedling growth are the most vulnerable periods in the seed life process and germination is easily disturbed by numerous stressors that in turn, affect seedling growth and final yield and quality (Smolikova and Medvedev, 2022). Therefore, successful seed germination and healthy seedling growth is a necessity for sustained crop production.

Under salt stress, plants reduce the toxic effects of salt on plants through numerous mechanisms including activation of antioxidant enzymes and synthesis of antioxidant compounds, ion homeostasis, biosynthesis of osmoprotectants and hormonal regulation (Sharaya et al., 2023). Microorganisms in the soil can also improve plant growth under salt stress via of the action of phytohormones, osmotic regulators, antioxidant enzymes and exopolysaccharide production (Choudhary et al., 2022). Additionally, seeds can be coated with microorganisms to improve performance and reduce production costs. This has been an effective method to reduce damage from abiotic stressors (Maity et al., 2019; Soumare et al., 2020). Microbial seed coating can ensure healthy seeds, improve germination, provide beneficial microorganisms that contributes to sustainable agriculture (Pedrini et al., 2017; Rocha et al., 2019; Zvinavashe et al., 2021). For instance, rhizobacteria such as *Rhizobium tropici* CIAT 899 have been used with the bean (*Phaseolus vulgaris*) as a model system to demonstrate that rhizobacteria delivered to the soil after coating dissolution are able to infect seedling roots, form root nodules, enhance yields, boost germination, and mitigate the effects of soil salinity (Zvinavashe et al., 2019). However, these coatings are insufficient to ensure microbial survival, are not stable for long term storage and are susceptible to environmental influences (Ma et al., 2010).

Microencapsulation is a novel technology to encapsulate microorganisms on a microscopic scale for their immobilization and protection. These provide a microenvironment that is less disturbed by adverse external environmental factors and can preserve viability (Kim et al., 2012). One such capsule material is the non-toxic and biocompatible alginate (Krasaekoopt et al., 2003) and improved microbial survival under salt stress was enhanced when applied as a seed coating agent to encapsulate *Pseudomonas fluorescens* VUPF506. This combination also successfully improved disease resistance and yield of potatoes (Fathi et al., 2021). Cotton seeds encapsulated with *Bacillus subtilis* SL-13 significantly improved cotton growth indicators including germination rate, fresh and dry weights and increased levels of the antioxidant enzymes peroxidase (POD) and superoxide dismutase (SOD) and accordingly, significantly decreased malondialdehyde (MDA) content (Tu et al., 2016).

The wheat (*Triticum aestivum* L) is one of the three major food crops in the world and is sensitive to salt levels. High saline soils retard its growth and delay development so that quality and yield suffer (Huang et al., 2023). The marine kelp (*Laminaria japonica*) is a widely distributed oceanic brown alga that is fast growing and highly adaptable to changing environmental conditions. Microorganisms associated with kelp are highly salt tolerant and can adapt to saline areas in coastal regions. Kelp-associated bacteria were able to mitigate the effects of salt stress on the growth and yield of rice (Rima et al., 2018). Therefore, microorganisms from kelp have potential uses as microbial seed coatings.

The objective of this study was to extract bacteria from kelp, encapsulate them by endogenous emulsification and prepare them as microbial seed coating. We then evaluated germination, biomass, chlorophyll content and indicators of oxidative stress in plants cultivated in the presence of 100 mM/L NaCl. This study provides information for the successful application of microbial microencapsulated seed coatings.

## Materials and methods

### Microorganisms and culture medium

Bacteria were isolated from fresh kelp as previously reported with some modifications (Li et al., 2022). In brief, 10 g of fresh kelp were cut into 0.5 cm^2^ pieces and added to 90 mL enrichment medium (5 g (NH_4_)_2_SO_4_, 15 g NaCl/L pH 7.5) in a 500 mL triangular bottle and incubated at 30°C with shaking at 180 rpm for 3 d until the plant pieces were visibly degraded. Samples (10 mL) were then transferred to another 500 mL bottle containing 90 mL of the same medium and incubated under the same conditions. The transfer and incubation process were again repeated for a total of 3 times. Dilutions of the enriched bacterial broth were plated on 1.5% agar isolation plates containing 1 g peptone, 1 g yeast paste, 5 g (NH_4_)_2_SO_4_, 15 g NaCl, pH 7.5 and incubated at 30°C for 1-2 d. Colonies were picked and purified 3 × using streak-plating until a single pure colony was obtained. The isolates were preserved and submitted to the commercial company Allwegene (Beijing, China) for 16S rDNA sequencing to identify the bacterial species.

### Preparation of the microcapsules

**Figure 1:**
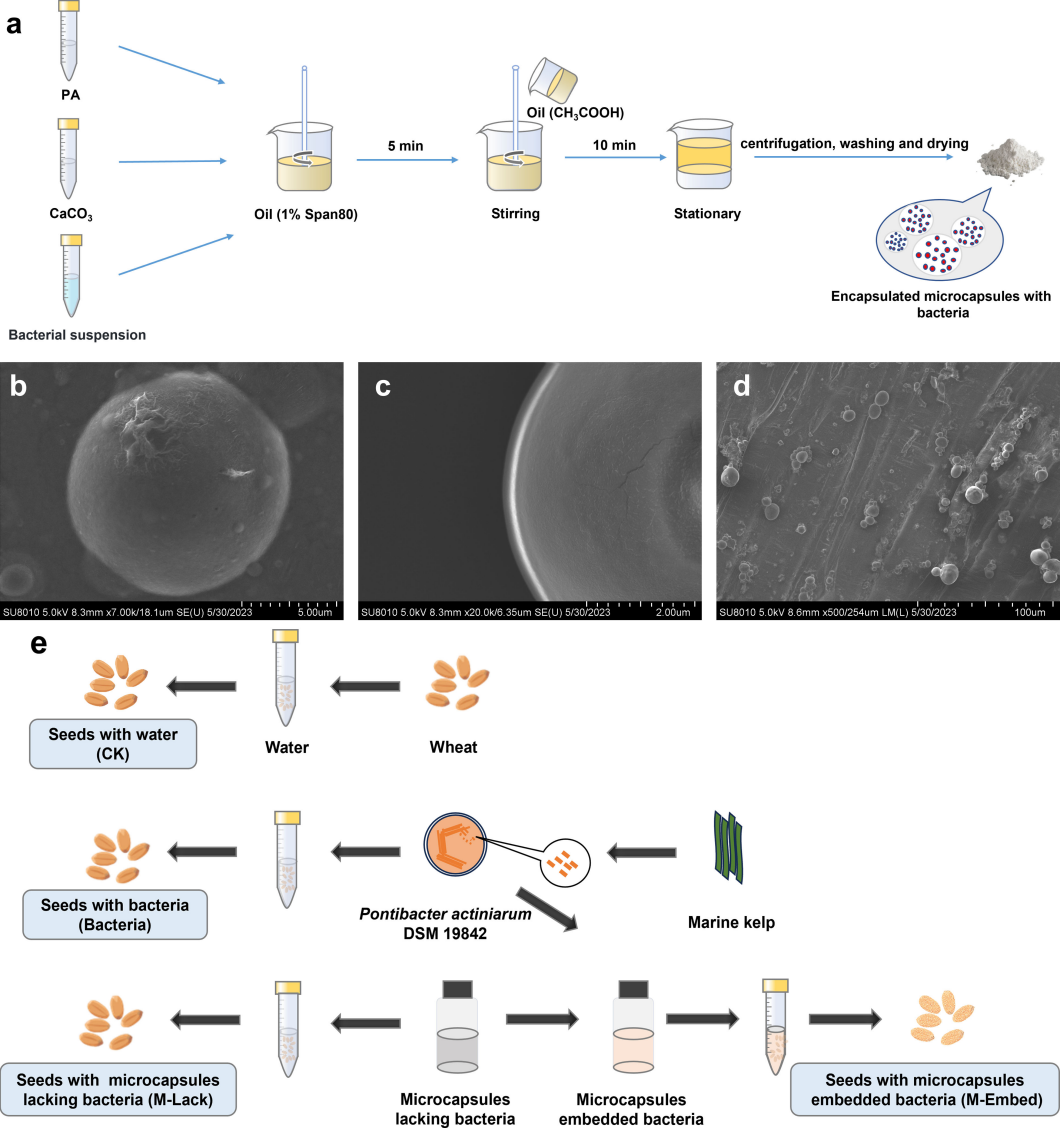
**(A)** Schematic of the process of preparing microcapsules by emulsion and internal gelation. **(B, C)** SEM of microbeads loaded with *P. actiniarum* DSM 19842. **(D)** SEM of wheat seeds dipped in microcapsules. **(E)** Illustration of the seed coating protocol.

Bacterial microencapsulation was performed by a modification of the emulsion method as reported previously (Qi et al., 2019). Briefly, 50 mL of sterile 2% (w/v) potassium alginate and 5 mL of bacterial suspension (10^8^ colony-forming units (CFU) mL^-1^) were mixed with 0.5 g calcium carbonate and homogenized. The mixture was dispersed into a 100 mL soybean oil phase that contained 1% (w/v) Span 80 and then was emulsified by stirring at 400 rpm for 5 min. Glacial acetic acid (0.25 mL) in 50 mL soybean oil was then added and stirring was continued for 10 min. After standing and stratifying, the aqueous (lower) phase was removed and centrifuged, washed with phosphate buffer and dried to obtain microcapsules. The oil layer on the top phase was harvested by aspiration and centrifuged for the next use. Hollow microcapsules lacking bacteria were harvested and dried in the same way and used as a control (**Figure 1A**).

### Calculation of bacterial embedding levels

1.0 g of microcapsules was weighed into 9 mL of NaH_2_PO_4_ solution (0.1 mol/L, NaH_2_PO_4_, pH 7.0) and shaken at a constant temperature of 37°C for 1 h to dissolve the microcapsules and release the encapsulated bacteria. After a series of 10-fold gradient dilutions, the appropriate dilution of the bacterial suspension was spread on the agar growth medium and incubated at 37°C for 48 h to determine the number of colony-forming units (CFU). The embedding levels were calculated as follows (Fareez et al., 2015):


$$\text{Embedding}\left(\%\right){\text{=N}}^{\text{u}}{\text{/ N}}^{\text{t}}\times 100$$

where N^u^ represented CFU following capsule breakage and N^t^ the CFU of the bacterial solution prior to encapsulation.

### Scanning electron microscopy

Microcapsule morphology was observed using a JSM-7401F scanning electron microscope (SEM) (JEOL, Tokyo, Japan). Aqueous dispersions of the samples were dropped onto clean silicon wafers and air-dried and plated with platinum using an ETD-800 sputter coater (Beijing, China). The sample area not covered by the carrier was selected to observe the morphology of the nanoparticles. Statistical particle sizes based on SEM images were calculated using Nano Measurer (https://nano-measurer.updatestar.com/en) software.

### Determination of microcapsule particle size

Microcapsule particle sizes were measured using a Mastersizer 2000 laser particle size analyzer (Malvern Panalytical, Malvern, UK). The magnitude of the span value indicated the degree of dispersion of the particle size and was determined as follows (Lemos et al., 2017):


$$Span\;=\;\left(D_{90}-\;D_{10}\right)\;/\;D_{50}$$

where D_10_, D_50_ and D_90_ represented the particle size values corresponding to 10%, 50% and 90% on the cumulative percent particle size distribution curve.

### Microcapsule storage stability

The microbial microcapsules prepared by emulsion and internal gelation were placed at room temperature (20-25°C) for storage and samples were taken every 10 d for CFU counting as per above.

### Wheat planting experiments

The wheat (*Triticum aestivum* L., Jimai 22) seeds were sterilized with 1% NaClO for 10 min and then rinsed 3 × with dH_2_O. Full-grained seeds were planted in pots with 50 seeds per pot (diameter 15cm, height 13cm). The salt stress experiment utilized the same concentration gradient of salt stress as that used in (Wahid et al., 2021). In the experiment, one third height of the pots with substrate were put into a solution of 100 mM/L NaCl (11.2 dS/m), through capillary movement, the NaCl uniformly distributed in the pots. The seeds were then planted. Seedlings were grown at 25°C or 20°C (day or night) with a 12 h photoperiod at a light intensity of 240 μmol m^-2^ s^-1^.

The seed coating experiments included (i) Wheat seeds with water (CK), (ii) Wheat seeds with bacteria (Bacteria), (iii) Wheat seeds with microcapsules lacking bacteria (M-Lack) and (iv) Wheat seeds with microcapsules embedded bacteria (M-Embed) (**Figure 1E**). Four replicates were used for each treatment, all of which were randomly arranged and rotated periodically to minimize the effects of environmental heterogeneity.

### Plant growth indicators

Wheat germination rate (%) was recorded after 7 d treatment under salt stress. The germination rate was calculated as:


G = a / b × 100

where G is the germination rate (%), parameter a is the number of germinating seeds, and b is the total number of seeds from the germination test.

4 plants were randomly selected from each treatment. The plants were divided into above-ground and below-ground parts from the rootstock union and the above-ground parts were measured for fresh weight. Above-ground dry weight was measured after drying the samples in an oven at 70°C for 48 h. The intact wheat roots were scanned and analyzed using WinRHIZO (Regent Instruments, Québec, Canada) to quantify root morphology using the protocol of the manufacturer.

### Plant physiological indicators

Following 7 d under salt stress, 4 plants were randomly selected from each treatment to determine the physiological indicators. The content of physiological indicators is calculated by measuring the absorbance at the corresponding wavelength. Absorbance was measured three times per sample. SOD (absorbance at 450 nm), POD (absorbance at 470 nm), CAT (absorbance at 510 nm), PRO (absorbance at 520 nm), MDA (difference between 600 and 520 absorbance) and H_2_O_2_ (absorbance at 415 nm) levels were determined using commercial kits (Suzhou Grace Biotechnology, Suzhou, China) (He et al., 2022). Seven days after planting, wheat samples were collected, rapidly frozen in liquid nitrogen and ground into fine powders (40 Hz, 1 min). A total of 100 mg of each sample was dissolved in Kit-provided PBS. The mixture was then vortexed for 10 min and centrifuged for 5 min (12000 rpm, at 4°C). The supernatants were used for enzyme activity assessments using a 96-well Microplate Reader (Thermo Scientific, Pittsburg, PA, USA) at specific wavelengths as described in the kit instructions. Chlorophyll (Chl) was determined using a model TYS-B portable chlorophyll meter (Zhejiang TOP Cloud-Agri Technology, Zhejiang, China). Three replicates were carried out for each treatment.

### Statistical analysis

All the data were analyzed using SPSS 21 (IBM. Armonk, NY, USA). The data were analyzed using two-tailed Student’s t test for single comparisons. The differences between experimental treatments were evaluated by the least significant difference test (LSD) at *p* < 0.05. GraphPad (GraphPad, Software, USA) was used to create the graphs. Mean values and standard errors (SE) are presented in figures and tables. Structural equation modeling was used to explore possible pathways for plant stress tolerance, plant root growth, H_2_O_2_ and MDA effects (shoot/root ratios) on fresh and dry weight. We chose the model with the smallest AIC value for comparisons.

## Results

### Strain identification and characterization

Bacteria associated with kelp plants were isolated by enrichment and then purified on agar plates. One species was isolated and selected and identified as the type strain (*Pontibacter actiniarum* DSM 19842) originally identified in the Pacific Ocean in Rudnaya Bay, Russia. *P. actiniarum* is an aerobic Gram-negative, pink-pigmented marine bacterium with gliding mobility of the phylum *Bacteroides* (https://doi.org/10.1099/ijs.0.63819-0).

### Microencapsulation analysis

*P. actiniarum* microcapsules that were prepared using the emulsion and internal gelation method were present as smooth, spherical and individual microcapsules with no apparent pores (**Figures 1B, C**). The microcapsule average particle size was 10.6 µm and concentrated in the range of 5.5-15.9 µm. The particle size distribution range span was 1.04 and indicated a uniform particle size distribution. The microcapsules were applied to wheat seeds by dipping and adherence to the seeds was apparent in SEM photomicrographs (**Figure 1D**).

### Bacteria microcapsule embedding and stability

The bacterial embedding score was examined by comparing bacterial CFUs of solutions used for encapsulation with those following bacteria release from the microcapsules. The encapsulation rate reached 65.4%. Survival rate of *P. actiniarum* in the microcapsules following 60 d of storage at room temperature (20-25°C) was 73.1%, while survival rate of those not microencapsulated were 49.3%, and increased by 22.4%.

### Germination and seedling development

**Figure 2:**
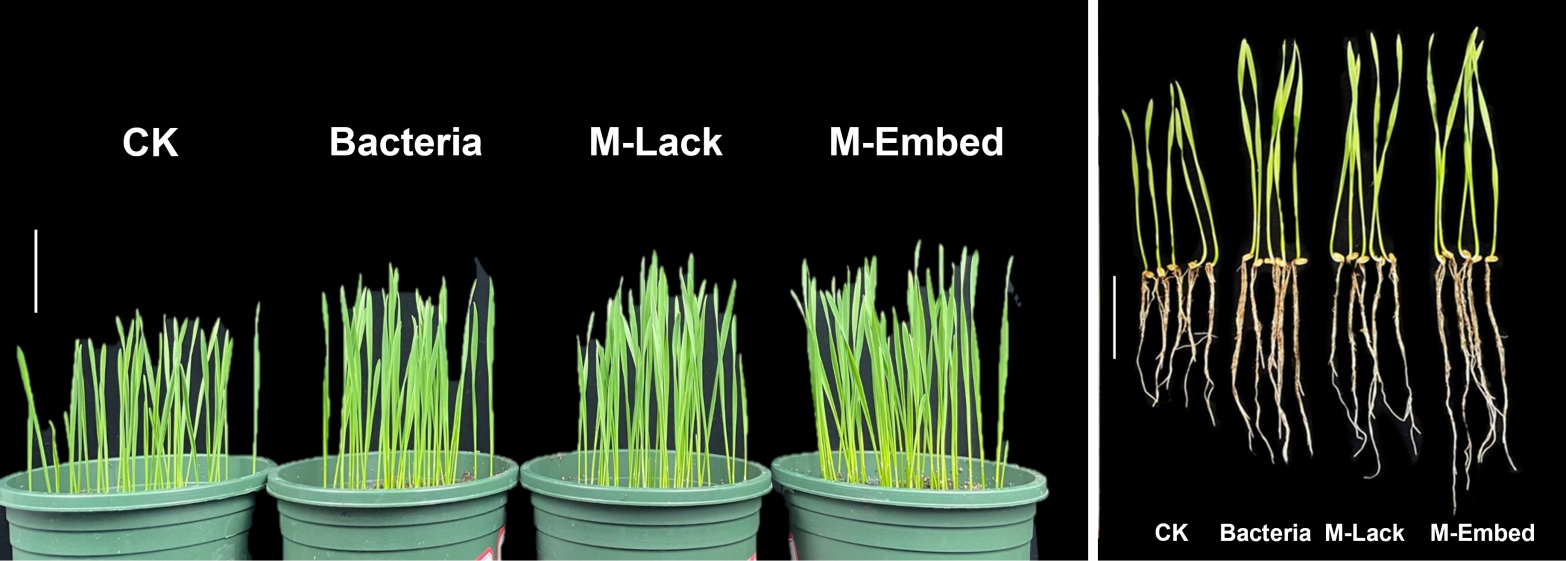
Photographs of wheat seedlings grown for 7 days. Wheat seeds with water (CK); Wheat seeds with bacteria (Bacteria); Wheat seeds with microcapsules lacking bacteria (M-Lack); Wheat seeds with microcapsules embedded bacteria (M-Embed). (Scale bar is 5 cm).

**Figure 3:**
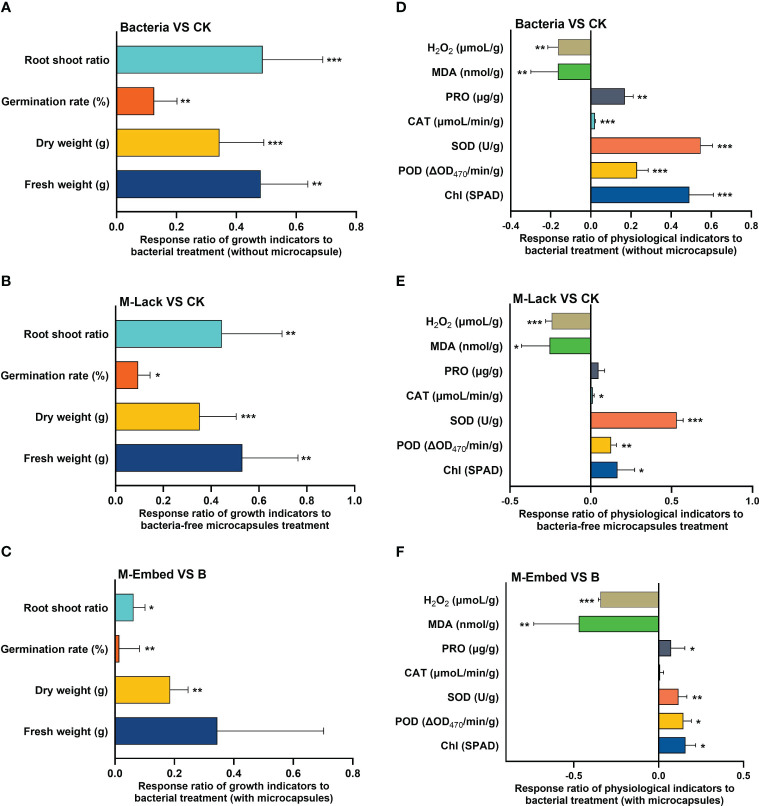
Response ratio calculations for root/shoot, germination (%), dry/fresh weights for plants for the groups **(A)** Bacteria, **(B)** M-Lack and **(C)** M-Embed. Response ratios for Chl, POD, SOD, CAT, PRO H_2_O_2_ and MDA for **(D)** Bacteria, **(E)** M-Lack and **(F)** M-Embed. Superoxide dismutase (SOD), peroxidase (POD), catalase (CAT), proline (PRO), malondialdehyde (MDA), hydrogen peroxide (H_2_O_2_) and Chlorophyll (Chl). TRL, Total root length; TRA, total root area; ARD, average root diameter; TRV, total root volume; RHL, root hair length. *p<0.05, ** *p*<0.01, *** *p*<0.001.

Under salt stress, seed germination is considered the key point throughout the plant growth period and after that, plants can cope with salt stress in the environment through other strategies. Wheat seeds treated with Bacteria, M-Lack and M-Embed all had higher germination rates (84%, 82% and 90% respectively) than the controls (average of 73%). We measured the response ratios for the 4 experimental treatments (**Figure 2**). Bacteria, M-Lack and M-Embed all displayed significant increases in germination compared to the CK while M-Embed displayed a significant increase in germination compared to group Bacteria. In addition, Bacteria and M-Lack displayed a significant increase in dry and fresh weight as well as in the root/shoot ratio compared to CK while M-Embed possessed a significant increase in the dry weight and root/shoot ratio compared with Bacteria (**Figure 3**).

The increases in the root/shoot ratio were correlated with the presence of bacteria and microcapsules on the wheat root system. M-Lack and Bacteria displayed significant increases in total root length (TRL), total root area (TRA), total root volume (TRV) and root hair length (RHL) compared to CK. In addition, M-Embed plants displayed significant increases in TRL, TRA, TRV and RHL and a significant decrease in mean root stem compared to Bacteria (**Table 1**).

**Table 1:** Mean and SD of root growth factors under different coating treatments of wheat seeds.

Treatments	TRL (cm)	TRA (cm^2^)	ARD (mm)	TRV (cm^3^)	RHL (cm)
CK	97.04 ± 2.99a	9.86 ± 0.62a	0.2132 ± 0.0071a	0.0797 ± 0.0074a	31.49 ± 1.38a
Bacteria	137.44 ± 3.82b	14.24 ± 0.44b	0.2541 ± 0.0403ab	0.1175 ± 0.0055b	43.59 ± 3.58b
M-Lack	135.48 ± 5.02b	13.94 ± 0.44b	0.2919 ± 0.0015b	0.1140 ± 0.0030b	44.11 ± 1.35b
M-Embed	160.32 ± 5.58c	17.92 ± 0.79c	0.2330 ± 0.0040c	0.1593 ± 0.0094c	56.98 ± 1.24c

TRL, Total root length; TRA, total root area; ARD, average root diameter; TRV, total root volume; RHL, root hair length. ^a-c^ Different letters indicate significant statistical differences (p < 0.05, Tukey’s test).

### Physiological activity

Our response ratio analysis indicated a significant increase in the physiological indices Chl, POD, CAT and SOD in Bacteria and M-Lack compared to CK as well as a significant increase in PRO in Bacteria. There was also a significant decrease in MDA and H_2_O_2_ in both Bacteria and M-Lack compared with CK. The physiological index levels of Chl, POD, SOD and PRO were significantly higher in M-Embed than in Bacteria while MDA and H_2_O_2_ were significantly lower. (**Figure 3**). In addition, two-factor ANOVA tests indicated significant interactions between M-Lack and Bacteria for SOD (**Table 2**).

**Table 2:** Repeated measurement ANOVA for coatings for the indicated indices for wheat.

Variables	Df	Bacteria	M-Lack	Bacteria × M-Lack
Wheat growth indicators				
Root shoot ratio	1	47.03***	32.81**	15.18**
Germination rate	1	13.78**	5.29*	ns
Dry weight	1	74.38***	96.69***	ns
Fresh weight	1	77.76***	27.31***	6.00*
Wheat physiological indicators				
Chl	1	111.73***	12.92**	ns
POD	1	101.68***	33.46***	ns
SOD	1	344.97***	308.29***	86.67***
CAT	1	10.10**	ns	ns
PRO	1	56.09***	6.49*	ns
MDA	1	13.67**	23.61***	ns
H_2_O_2_	1	60.59***	116.17***	ns
Wheat root growth				
TRL	1	213.26***	188.43***	12.13**
TRA	1	202.28***	173.98***	ns
ARD	1	ns	7.81*	23.47***
TRV	1	151.53***	127.32***	ns
RHL	1	137.65***	149.55***	ns

Effects of wheat seeds with bacteria (Bacteria), wheat seeds with microcapsules lacking bacteria (M-Lack) and wheat seeds with microcapsules embedded bacteria (M-Embed). Root shoot ratio, Germination rate, Dry weight, and Fresh weight), wheat physiological indicators (Chl, POD, SOD, CAT, PRO, H_2_O_2_ and MDA), and wheat root growth (TRL, TRA, ARD, TRV, and RHL) for repeated measures ANOVA results F values. See **Figure 3** for abbreviations. * p < 0.05, **p < 0.01, *** p < 0.001.

**Figure 4:**
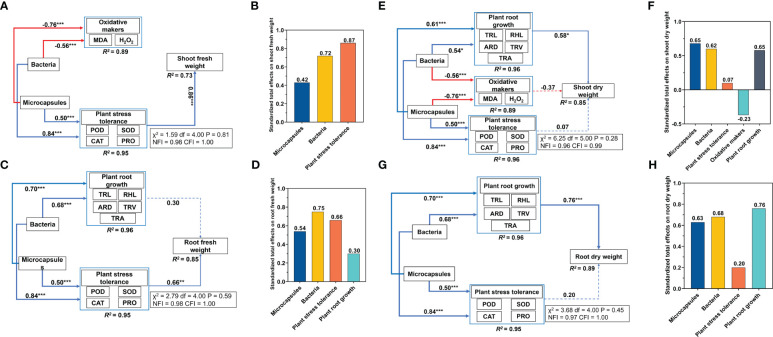
Results of the structural equation modeling. **(A, C, E, G)** standardized total effects of bacteria and **(B, D, F, H)** microcapsules on shoot/root fresh and dry weights. See **Figure 3** for abbreviations. Red lines, negative correlations; blue lines, positive correlations. Standardized path coefficients are described by the numbers on the lines. The proportions of variance explained for each dependent variable in the model are shown at the bottom and details evaluating the models are shown in the boxes beside each figure. * p < 0.05, **p < 0.01, *** p < 0.001.

The increased levels of POD, CAT, SOD and PRO indicated that the plants were coping with adversity in a favorable way and the root system indicators displayed a similar pattern. We therefore used these factors to determine the plant stress tolerance using principal component analysis. Structural equation modeling indicated that bacteria and microcapsules had no direct effect on shoot and root fresh weights. Bacteria addition increased plant stress tolerance (0.84) and decreased oxidative makers content (0.56) and microcapsules increased plant stress tolerance (0.50) and decreased oxidative makers content (0.76). The plant stress tolerance increased shoot fresh weight (0.86) (**Figure 4A**). The standardized total effects of bacteria and microcapsules on shoot fresh weight were 0.72 and 0.42, respectively (**Figure 4B**). Overall, the indirect effects of bacteria and microcapsules worked by altering plant stress tolerance that explained 73% of shoot fresh weight (**Figure 4A**). Bacteria increased plant root growth (0.68) and plant stress tolerance (0.84) while microcapsules increased plant root growth (0.70) and plant stress tolerance (0.50). The plant stress tolerance increased root fresh weight (0.66) (**Figure 4C**). The standardized total effects for bacteria and microcapsules on root fresh weight were 0.75 and 0.54, respectively (**Figure 4D**). Overall, the indirect effects of bacteria and microcapsules through altered plant stress tolerance explained 85% of the root fresh weight (**Figure 4C**). The inferences of this model were that bacteria and microcapsules increased the fresh weight of wheat seedlings by indirectly increasing the plant resistance via elevated levels of POD, SOD, CAT and PRO.

Bacteria and microcapsules had no direct effect on the shoot dry weight and root dry weight. Bacteria increased plant root growth (0.54) and plant stress tolerance (0.84) and decreased oxidative makers content (0.56). Microcapsules increased plant root growth (0.61) and plant stress tolerance (0.50) and decreased oxidative makers content (0.76) while plant root growth increased shoot dry weight (0.58) (**Figure 4E**). The standardized total effects of bacteria and microcapsules on shoot dry weight were 0.62 and 0.65, respectively (**Figure 4F**). Overall, the indirect effects of bacteria and microcapsules through altered plant root growth explained 85% of the shoot dry weight (**Figure 4E**). Bacteria increased plant root growth (0.68) and plant stress tolerance (0.84) while microcapsules increased plant root growth (0.70) and plant stress tolerance (0.50). The standardized total effects of bacteria and microcapsules on the root dry weight were 0.68 and 0.63, respectively (**Figure 4H**). Overall, the indirect effects of bacteria and microcapsules that acted via altering plant root growth explained 89% of the root dry weight (**Figure 4D**). The bacteria and microcapsules treatments indirectly increased plant root growth that included TRL, RHL, ARD, TRV and TRA as well as plant dry weight.

## Discussion

Germination and seedling stages are critical points in the crop growth cycle and are vulnerable to environmental stressors (Nakhla et al., 2021; Tarchoun et al., 2022). Seed coating is a technology that covers the surface of seeds with a material that improves germination, crop growth and yield and has become an effective way to reduce production costs in precision agriculture by requiring only a small amount of microbial coating (Sohail et al., 2022). Bacteria seed coating can form a protective film on the surface of seeds and enhances plant tolerance and promote plant growth (Rocha et al., 2019). However, traditional microbial coating agents suffer from poor adhesion, low survival and storage stability resulting in reductions in bacteria numbers and activity (Afzal et al., 2020).

In the current work, we found that seeds embedded with microencapsulated bacteria under salt stress conditions performed better than control counterparts in terms of growth and physiological indices. The encapsulated bacteria were thus present in a more favorable microenvironment and a protective effect was achieved using an alginate and bacterial coating. Microencapsulation of the bacteria improved germination and conferred significant seedling viability. The primary capsule material calcium alginate worked with the bacteria for the best effect on plant growth although individual action of each also promoted plant growth.

The bacterium (*P. actiniarum* DSM 19842) used in these experiments was obtained from fermentation of kelp collected from the ocean. The closely related *Pontibacter actiniarum* KMM 6156T used in previous studies possessed peroxidase and oxidase activities that could minimize oxidative damage caused by salt stress (Chhetri et al., 2019). It has been shown that alginate is a major compound of macroalgae and as such an important carbon and energy source for marine bacteria (Jouanneau et al., 2021).

Calcium alginate has a mitigating effect on plants grown in salt-stressed environments. This compound can promote root development and growth and increase root absorption area. These effects increase water and nutrient absorption counteracting the effects of salt stress (Xu et al., 2003). Calcium ions in calcium alginate also can act to regulate ionic balance in plants. Salt stress will lead to an increase in Na^+^ content, thus reducing the absorption of K^+^, Mg^2+^ and Ca^2+^. The addition of Ca^2+^ can attenuate the cytotoxicity caused by Na^+^ ions under salt stress and promote ionic homeostasis (Guo et al., 2021). In addition, calcium alginate has antioxidant capacities. Salt stress can lead to excessive production of reactive oxygen radicals in plants triggering oxidative damage (Mangal et al., 2023). The natural oxidizing substance of calcium alginate can assist plants in free radical scavenging to reduce oxidative damage (Kumari and Bhatla, 2021). Alginate may also contain small amounts of potassium alginate and K^+^ has equally positive effects in maintaining ionic balance (Kumari and Bhatla, 2021), improving the antioxidant capacity of plants (Rady et al., 2022) and promoting plant root development (Bojórquez-Quintal et al., 2016).

Salt stress affects plant survival and crop yield by inhibiting seed water uptake (Zhu et al., 2019), interfering with metabolic activities within the seed (Zou et al., 2020) and inhibiting seed enzyme activities (Gou et al., 2020) leading to decreased levels of germination. In our experiments, M-Embed had significant increases in germination under salt stress and these plants displayed significantly increased values for TRL, TRA, TRV and RHL compared to the other treatments. Salt stress can damage the osmotic balance of plant cells and lead to water loss while these significant changes in root-to-crown ratios and root structure can increase the ability of plants to access available water and nutrients (Li et al., 2021). Therefore, in terms of growth indicators, M-Embed reduced the damage caused by salt stress on plant growth by increasing germination and promoting root growth. The structural equation modeling demonstrated that the use of bacteria and microcapsules can play an important role in improving plant root growth and increasing plant dry weight (**Figure 4**).

The protection of plant photosynthetic mechanisms contributes to the ability of plants to resist the generation of reactive oxygen species (ROS) radicals under salt stress as well as to participate in the scavenging of preexisting ROS (Tian et al., 2022). The experiments demonstrated leaf chlorophyll content in M-Embed under salt stress was greater than for the other treatments and would assist plants in maintaining a normal metabolism. Previous studies have indicated that bacteria promote photosynthetic activity in plants under salt stress possibly due to increased levels of the plant growth regulators indole acetic acid (IAA) (Cheng et al., 2022) and 1-aminocyclopropane-1-carboxylate (ACC) deaminase involved in ethylene regulation (Misra and Chauhan, 2020) as well as with extracellular polymeric substances (EPS) (Amna et al., 2019). These compounds can also increase chlorophyll levels. Alginate therefore can play an important role in plant-microbe interactions (Fathi et al., 2021).

Under salt stress, ROS accumulates and produces oxidative damage to plant cells. H_2_O_2_ is one of the most abundant reactive ROS in cells and H_2_O_2_ content plays an important role in plant salt tolerance (Wu et al., 2018). SOD, CAT and POD improve plant adaptation by reducing the oxidative damage to cells by participating in ROS scavenging (Santos et al., 2018). We found that antioxidant enzyme levels in wheat seedlings for M-Embed were higher than for the other treatments while the H_2_O_2_ content was lower than in the other treatments. Thus, wheat with microbial microcapsules coating showed better performance than other treatments in terms of antioxidant defense mechanisms.

Osmoregulation is a central part of the physiological mechanism of plant response to salt stress. Under salt stress, osmoprotectants such as free proline, glycine betaine and other amino acids are formed (Suprasanna et al., 2016). These assist cells to maintain normal osmoregulation and cell membrane stability, ultimately improving plant growth and development (Suprasanna et al., 2016). Therefore, PRO was one of the key factors in the osmoregulatory function of plants. We found that M-Embed had the highest PRO content compared to the other treatments. This could be a mechanism to alleviate the problem of osmotic accumulation caused by salt stress. Structural equation modeling indicated that bacteria and microcapsules affect plant stress tolerance and indirectly increase plant dry weight. Thus, the changes in antioxidant enzymes and proline induced by M-Embed were important factors that increase plant dry weight (**Figure 4**).

One of the main consequences of salt stress is excess ROS in plant cells leading to lipid peroxidation that damages the cell membrane and increases MDA in leaves (Sun et al., 2010). The presence of bacteria can inhibit oxidative damage thereby enhancing plant cell stability decreased levels of ROS under salt stress conditions (Bharti et al., 2013). The lowest levels of the oxidative markers (MDA and H_2_O_2_) in M-Embed were also an indicator of decreased oxidative damage.

## Conclusions

This promising seed coating improved germination, promoted root growth, increased levels of enzymatic antioxidants and osmoprotectants and reduced oxidative stress markers. Based on these results, microencapsulation of *P. actiniarum* for use as seed coatings can reduce the negative effects of salt stress on plant growth. This study provides new insights into the application of microcapsules in functional seed coating. Moreover, future research is needed to confirm the interaction process between the microbe and plant relationship.

## Data availability statement

The original contributions presented in the study are included in the article/supplementary material. Further inquiries can be directed to the corresponding authors.

## Ethics statement

The manuscript presents research on animals that do not require ethical approval for their study.

## Author contributions

MG: Conceptualization, Data curation, Formal Analysis, Methodology, Project administration, Writing – original draft. JH: Formal Analysis, Methodology, Writing – review & editing. MK: Formal Analysis, Methodology, Writing – review & editing. QH: Formal Analysis, Methodology, Project administration, Writing – review & editing. YJ: Formal Analysis, Methodology, Project administration, Writing – review & editing. JS: Writing – review & editing, Methodology. WH: Funding acquisition, Supervision, Validation, Writing – review & editing. GL: Funding acquisition, Supervision, Validation, Writing – review & editing, Investigation, Methodology, Resources.
